# Prospects of Curcumin Nanoformulations in Cancer Management

**DOI:** 10.3390/molecules27020361

**Published:** 2022-01-07

**Authors:** Hilda Amekyeh, Enas Alkhader, Rayan Sabra, Nashiru Billa

**Affiliations:** 1 Department of Pharmaceutics, School of Pharmacy, University of Health and Allied Sciences, Ho PMB 31, Ghana; hamekyeh@uhas.edu.gh; 2 Faculty of Pharmacy, Middle East University, Amman 11831, Jordan; ealkhader@meu.edu.jo; 3 Department of Pharmaceutical Sciences, University of Connecticut, Storrs, CT 06269, USA; rayan.sabra@uconn.edu; 4 Pharmaceutical Sciences Department, College of Pharmacy, QU Health, Qatar University, Doha P.O. Box 2713, Qatar

**Keywords:** curcumin, cancer, nanoparticles, drug delivery, chemotherapy, bioavailability

## Abstract

There is increasing interest in the use of natural compounds with beneficial pharmacological effects for managing diseases. Curcumin (CUR) is a phytochemical that is reportedly effective against some cancers through its ability to regulate signaling pathways and protein expression in cancer development and progression. Unfortunately, its use is limited due to its hydrophobicity, low bioavailability, chemical instability, photodegradation, and fast metabolism. Nanoparticles (NPs) are drug delivery systems that can increase the bioavailability of hydrophobic drugs and improve drug targeting to cancer cells via different mechanisms and formulation techniques. In this review, we have discussed various CUR-NPs that have been evaluated for their potential use in treating cancers. Formulations reviewed include lipid, gold, zinc oxide, magnetic, polymeric, and silica NPs, as well as micelles, dendrimers, nanogels, cyclodextrin complexes, and liposomes, with an emphasis on their formulation and characteristics. CUR incorporation into the NPs enhanced its pharmaceutical and therapeutic significance with respect to solubility, absorption, bioavailability, stability, plasma half-life, targeted delivery, and anticancer effect. Our review shows that several CUR-NPs have promising anticancer activity; however, clinical reports on them are limited. We believe that clinical trials must be conducted on CUR-NPs to ensure their effective translation into clinical applications.

## 1. Introduction

According to Global Cancer Incidence, Mortality and Prevalence (GLOBOCAN) 2020, over 19.3 million new cancer cases were diagnosed globally in 2020, with nearly 10.0 million deaths attributed to this statistic [1]. Specifically, lung cancer is the prominent cause of death from cancer in men, while breast and cervical cancers are the primary causes of cancer death in women. Cancer originates when cells in the body start to grow out of control and typically develop slowly over several years. Thus, cells in almost any part of the body can potentially develop into a cancer and can spread to other areas of the body.

The prognosis and treatment options for cancer mainly depend on the stage of the cancer, tumor recurrence, and the patient’s general health. The four prevalent cancer treatment procedures are surgery, chemotherapy, radiotherapy, and targeted therapy [2]. Surgery is the first therapeutic approach and primary procedure. Usually, surgery is combined with chemotherapy and/or radiation therapy to avoid residual tumor recurrence. In radiotherapy, energy and X-rays produced by a linear accelerator are employed to destroy cellular DNA and inhibit cell proliferation [3]. On the other hand, chemotherapy relies on cytotoxic drugs to inhibit the growth of cancer cells and kill them [4]. Chemotherapy is either administered before surgery to shrink tumors, or after surgery as adjuvant chemotherapy. Due to the limited efficacy of non-selective chemotherapeutic drugs, targeted therapy has evolved as a rational option. In targeted therapy, drugs are targeted to specific receptors present on cancer cells or in structures related to cancer growth, such as blood vessels. Additionally, cancer cell proliferation and metastasis are blocked via the inhibition of specific mediators such as epidermal growth factors (EGFs) [5]. However, the effectiveness of targeted therapy depends on the release of the therapeutic agent at the cancer target site, as well as minimizing off-target side effects to normal tissues [6].

Regardless of the advanced innovations in cancer therapy, treatment remains arduous. For instance, surgery is affiliated with detrimental harm to adjacent organs and tissues, discomfort, infections, and relapse [7,8,9]. While chemotherapy, the typical cancer treatment option, whether administered as a neoadjuvant, adjuvant, or sole therapy, demonstrates severe side effects, involving fatigue, sores in the mouth and throat, nausea, vomiting, and blood disorders [10,11,12]. Radiation therapy also manifests a wide range of side effects akin to skin changes, fatigue, and diarrhea, among others, that disturb the well-being of the patients. Even though targeted therapy has emerged as an approach to conquer the lack of specificity in conventional chemotherapy, there are potential risks and challenges associated with this novel strategy. For example, some cancer cell types develop resistance to drugs over the treatment course, thereby rendering the targeted therapy ineffective by driving the drug out of the cancer cells and decreasing intracellular drug concentration [13]. Therefore, in most cases, targeted therapy is used in combination with chemotherapy; however, this strategy does not reduce the toxicity experienced with chemotherapeutic drugs.

To overcome the aforementioned constraints, chemotherapeutic agents from plants are becoming serious contenders as chemotherapeutic alternatives due to their manifestation of reduced toxicity to adjoining cells [14] while still providing potent anticancer effects in some cases. One such chemotherapeutic agent is curcumin (CUR), a major chemical constituent in turmeric, which has received much attention in the past decades because of its use in Indian and Chinese traditional medicine for centuries to treat a variety of conditions including infections, inflammation, and depression. It is also used as a spice. Thus, CUR is widely accepted by the public as it is derived from natural sources, all of which gives the perception that it is safe to use and likely to manifest fewer side effects when used therapeutically [15]. However, the systemic bioavailability following the oral administration of free CUR is poor due to the low solubility and rapid metabolism as stated earlier [16]. Aptly, extensive research has elaborated on the therapeutic potential of CUR against a range of cancers [17], but only through formulation intervention.

## 2. Curcumin (CUR)

### 2.1. General Background Information

CUR is a hydrophobic, orange-yellow, crystalline phytochemical derived from the rhizomes of turmeric (*Curcuma longa*), a plant which grows in the Indian subcontinent and tropical countries in South East Asia [13]. From ancient times, turmeric was therapeutically used to treat various respiratory conditions, liver disorders, abdominal pain, and many other ailments [18,19].

Later in the 13th century, turmeric was introduced to Europe as Indian saffron by Arab merchants and used as a colorant in foods, cosmetics, and textiles [20].

The primary source of turmeric is India, where about 80% of global turmeric is consumed [21]. Epidemiological studies attribute the low incidence of colon cancer in the Indian subcontinent to the chemo-preventive properties of diets rich in CUR [22].

**Figure 1:**
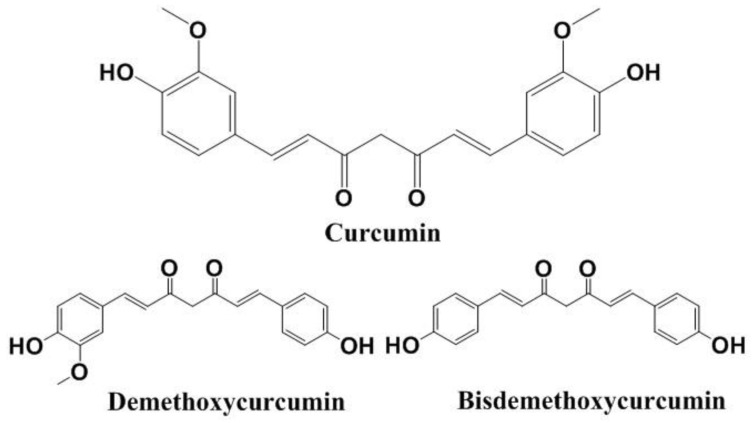
Chemical structures of curcuminoids.

The presence of a cluster of sesquiterpenes, such as *(S)-ar*-turmerone, zingiberene, β-turmerone, and curlone, and a variety of other volatile compounds (e.g., monoterpenes) in turmeric, gives its fragrance when used as a food seasoning [23]. The active constituents in turmeric are known as curcuminoids, with the major curcuminoid being a *bis*-α,β-unsaturated diketone. Apart from CUR, also called diferuloylmethane, the other two bioactive analogs are demethoxycurcumin (DMC) and bisdemethoxycurcumin (BDMC). The chemical structures of the three curcuminoids are presented in Figure 1.

CUR is considered the principal curcuminoid that exhibits most of the therapeutic activities in turmeric [24]. Moreover, the characteristic yellow color of turmeric is attributable to CUR [25]. 

The bright yellow color of curcumin turns red and yellow in basic and acidic media, respectively [26]. It also fluoresces under ultraviolet light. In aprotic solvents (e.g., ethyl acetate and acetone), CUR has a noticeable fluorescent intensity variation from 494 to 538 nm. In solvents such as alcohols and dimethyl sulfoxide (DMSO), the fluorescence shifts to 535–560 nm, while the fluorescence spectrum of CUR in cyclohexane is notably distinct with two fluorescence maximas at 446 and 470 nm [27,28,29].

The IUPAC name of CUR is 1,7-bis (4-hydroxy-3-methoxy-phenyl) hepta-1, 6-diene-3, 5-dione, with a molecular weight of 368.37 Da, melting point of 183 °C, and three pKa values of 7.80, 7.35, and 9.0 [14]. It demonstrates keto-enol tautomerism, with the predominant keto- form in acidic and neutral solutions, and a stable -enol form in alkaline media [30]. 

CUR is lipophilic and is thus insoluble in water, but soluble in organic solvents such as DMSO, methanol, acetone, and ethanol [22]. Furthermore, CUR is a potent H-atom donor at pH 3.0–7.0 and an electron donor at an alkaline pH, at which it dissociates to form feruloyl methane, ferulic acid, and vanillin [27,31,32]. The bis-keto form of CUR dominates under acidic and neutral conditions, whereas the enol tautomer prevails at pH values over 8.0 [14,20]. The stability of CUR is considered pH-dependent, with the least stability in acidic media [32].

Shen and Ji [33] found that CUR degrades to dihydroferulic acid and ferulic acid in cell culture media with 10% fetal bovine serum and in in vivo studies. Even though the mechanism of degradation is still vague today, it is thought to be through its α,β-unsaturated β-diketo moiety [34]. Fortunately, this constraint can be diminished by encapsulating CUR into either liposomes, cyclodextrin, lipids, surfactants, or polymeric nanoparticles (NPs) [29].

The most compelling and key rationale for the therapeutic use of CUR as an anticancer alternative is its extremely superior safety profile; thus, it is declared as GRAS (generally recognized as safe) by the United States Food and Drug Administration [18]. However, all the preclinical and clinical data from the oral administration of CUR have revealed that it manifests poor systemic bioavailability with high susceptibility to metabolic degradation, whereby only about 2.30 µg/mL of CUR was registered in serum levels after an oral administration of 10 g of CUR [20]. This shows that CUR undergoes extensive metabolic degradation prior to absorption within the intestine and liver, which minimizes its usefulness following oral intake. 

### 2.2. Pharmacological Properties of CUR

In the last few decades, there has been an increased interest by researchers in the therapeutic effects of CUR as a natural alternative to chemical drugs in the management of several ailments [34]. Indeed, CUR is reported to possess a variety of pharmacological activities, including antimalarial [35,36,37], antibacterial [38,39], antiviral [40,41,42], antifungal [43,44], antioxidant [45,46,47], anti-inflammatory [45,48,49], antidiabetic [50,51], anti-human immunodeficiency virus [52,53,54], and anticancer [38,39,55,56,57,58] activities.

### 2.3. Anticancer Properties of CUR 

Generally speaking, normal cells have a restrained balance between growth upholding and growth opposing signals [59]. Thus, the proliferation and differentiation of cells transpires only when required. However, this balance is disturbed in tumor cells, which show continuous cell proliferation, loss of differentiation, and programmed cell death. Consequently, a hyper-proliferative state of cells is attained, which presents as cancer [60]. Other peculiarities recognized in tumor cells include metastasis, angiogenesis, and apoptosis [61]. 

CUR has been extensively studied as a potential anticancer remedy, as well as a chemopreventive and direct therapeutic agent. The anticancer properties of CUR have been proven in vitro, in vivo, and in clinical studies. It is reported that the anticancer properties of CUR are exhibited via the inhibition of cell proliferation, induction of apoptosis, and devaluation of tumor load. 

#### 2.3.1. Effects of CUR on Transcription Factors

Specific transcription factors that are reported to be involved in the anticancer effect of CUR include nuclear factor kappa B (NF-κB) [62], activator protein-1 [63], early growth response-1 [64], peroxisome proliferator-activated receptor-γ [65], signal transducer and activator of transcription, hypoxia inducible factor-1 [66], β-catenin [67], NF-E2-related factor 2 [68], electrophile response element [69], and androgen receptor [70]. CUR is believed to modulate various signaling pathways, thereby contributing to the activation of the aforementioned transcription factors [71]. Thus, CUR is able to regulate cell proliferation, inflammation, metastasis, angiogenesis, and invasion [66].

#### 2.3.2. Effects of CUR on Growth Factors and Protein Kinase

CUR suppresses and downregulates the expression of several growth factors that contribute to the development of various cancers [72]. The activity of several tyrosine kinases increases due to mutations, which subsequently results in the malignant metamorphosis and metastasis of human cancers. CUR downregulates epidermal growth factor receptor (EGFR) activity and epidermal growth factor (EGF)-induced tyrosine phosphorylation of EGFR, eventually resulting in reduced protein kinase activity [73]. In addition, CUR suppresses the activities of protamine kinase, pp60c-src tyrosine kinase, autophosphorylation-activated protein kinase, and protein kinase C [74]. 

#### 2.3.3. Effects of CUR on Inflammatory Cytokines

NF-κB is activated during the initial stages of inflammation. Consequently, the production of the multifunctional cytokine tumor necrosis factor (TNF) is upregulated, which, in turn, activates the production of interleukin (IL)-1 [75]. The activation of both TNF and IL-1 promotes the expression of several genes and proteins that engender acute and chronic inflammation [76]. It has been reported that chronic inflammation and activation of inflammatory cytokines mediate tumorigenesis [75,77,78]. 

CUR shows a synergistic apoptotic effect when combined with TNF-related apoptosis-inducing ligand. It also shows anti-inflammatory activity by blocking the phosphorylation of IκBα and inhibitors of NF-κB. This results in the inhibition of NF-κB activation and, subsequently, TNF. Moreover, CUR inhibits TNF expression [79] as well as phorbol-methyl-acetate-induced TNF-α levels in various cells [80].

#### 2.3.4. Effects of CUR on Enzymes

CUR has regulatory effects on various enzymes associated with inflammation and cancer, including fatty acid synthase, ATP-citrate lyase (ACLY), stearoyl-CoA desaturase 1, and cholesterol O-acyl-transferase [78,81]. Additionally, CUR potently inhibits carbonyl reductase [82,83], downregulates the expression of other reductases in the aldo-keto reductase superfamily, and inhibits the transport of anthracyclines out of tumor cells [84].

Glutathione-S-transferase enzymes (GSTs) are reportedly involved in chemotherapy resistance in several cancer cell lines due to the methylation of GSTs [85]. CUR covalently binds to the catalytic thiolate of DNA methyltransferase 1, resulting in the blocking of DNA methylation of GSTs [86]. High levels of ACLY have been detected in various cancers such as breast, bladder, colorectal, lung, liver, prostate, and stomach tumors. ACLY activation is promoted by increased levels of glucose and insulin-like growth factors, which subsequently intervene in cancer progression [87]. However, CUR decreases the hepatic expression of ACLY [88].

Cyclooxygenase-2 (COX-2) expression is promoted by growth factors, inflammatory cytokines, oncogenes, carcinogens, and tumor promoters. However, COX-2 inhibitors are believed to aid in cancer prevention and treatment [85]. In a previous study, CUR was found to suppress COX-2 activity directly and selectively. Additionally, it inhibited bile acid and phorbol-ester-induced COX-2 expression and interferon-alpha-induced COX-2 activation [89]. These data clearly show that CUR has an anticancer effect.

## 3. Potential of Nanodrug Delivery Systems in Cancer Treatment

Nanodrug delivery has been proposed as the frontier for the effective delivery of anticancer agents and, hence, for cancer management. Oral nanodrug systems can be formulated to traverse the gastrointestinal epithelia effectively, and thus circumvent the metabolic constraints that the payload is subjected to within the gastrointestinal tract. According to the National Cancer Institute, nanotechnology has the potential of improving the current status of cancer detection, treatment, and prevention [90].

**Figure 2:**
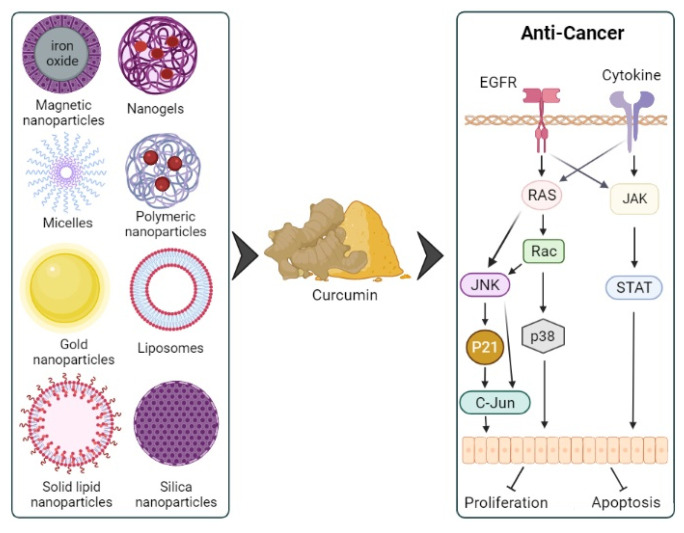
CUR-containing NP formulations with anticancer potential.

NPs have several characteristics ideal for the enhanced delivery of CUR in cancer management [91] (Figure 2). They exhibit a large surface area to volume ratio, high drug loading propensity, controlled drug release characteristics, and fairly good stability on storage [92]. NPs can be functionalized to deliver drugs specifically to cancer cells with minimal interaction with healthy tissue. Such targeting of NPs to cancer tissue is classified as either passive- or active-targeting [93]. The passive-targeting mechanism, also known as the EPR effect, occurs when NPs are in the size range of 10–100 nm, and, when in circulation, can selectively enter tumors through the surrounding leaky blood vessels and the interstitial space [94]. Owing to this EPR effect, NPs can also improve the safety and pharmacokinetic characteristics of active pharmaceutical ingredients [95]. Typically, NPs that employ the EPR effect to deliver drugs are intravenously administered since orally administered NPs have several hostile barriers to traverse within the gastrointestinal tract delivery in the systemic circulation. However, the active targeting of NPs to cancer cells entails specific ligand–receptor recognition and interaction on the cell surface [96]. 

Typically, cancer cells express higher levels of cell-surface receptors as compared to normal cells. This allows NPs conjugated with a targeting ligand to explicitly interact with them via receptor-mediated molecular recognition. There are several examples of CUR-NP delivery systems, which may be subdivided into organic and inorganic NPs [97]. Their particle size, surface charge (zeta potential, ZP), hydrophilicity/hydrophobicity, and composition, among other characteristics, can be tailored for a diverse array of applications [98]. However, the primary consideration when designing any drug delivery system is to control drug concentration within the therapeutic window and improve patient compliance in order to maintain effective treatment cycles with short recovery periods.

The following sections are a review of potentially useful CUR-containing NP preparations for the management of different cancers.

## 4. Prostate Cancer

### 4.1. Fibrinogen NPs

CUR-loaded fibrinogen NPs (CUR-FNPs) (size: 150–200 nm; ZP: −28 mV, encapsulation efficiency (EE): 90%) fabricated through chemical cross-linking with CaCl_2_ in a two-step coacervation process were found to be comparatively non-toxic to normal fibroblast L929 cells, but toxic to PC-3 prostate cancer cells, whereas free CUR showed no cytotoxicity [99]. The CUR-FNPs also showed a dose-dependent apoptotic effect on the cancer cells with significant internalization and retention within the cells. 

### 4.2. Cyclodextrin (CD)-Based NPs

Yallapu and colleagues [100] fabricated β-CD-CUR-NPs (52.6 nm) and found that uptake of the NPs by PC-3 cells was higher than that of free CUR. Similarly, the apoptosis rate was higher with the NPs, indicating the potential of the β-CD-CUR-NPs for managing prostate cancer. In addition, Ndong Ntoutoume and colleagues [101] prepared CUR-CD/cellulose nanocrystal complexes (206.8 nm) by ionic interaction with improved CUR solubility. The NPs showed a higher antiproliferative effect against PC-3 and DU145 cancer cells compared to free CUR. The half-maximal inhibitory concentration (IC_50_) values for the NPs after 48 h of treatment were 7.5 and 5.5 µM against the PC-3 and DU145 cells, respectively, as opposed to 10 and 18 µM, respectively, for free CUR. These findings are promising; however, in vivo studies are needed to confirm the potential of these CD-based CUR-NPs for managing prostate cancer.

### 4.3. Magnetic and Dendrimer-Based NPs

In the study conducted by Yallapu and colleagues [100], CUR-loaded magnetic and dendrimer-based NPs (8.6 and 37.4 nm, respectively) were also evaluated in PC-3 cells with similar cellular uptake findings. However, uptake was higher for the dendrimers due to their attachment to the cells. The considerably small sizes of these NPs hold potential for cancer management, but further studies are required.

### 4.4. Polymeric NPs

Yallapu and colleagues [100] also found that the uptake of CUR-NPs formulated with hydroxypropyl methyl cellulose (HPMC-CUR-NPs) and poly(lactic-co-glycolic acid) (PLGA-CUR-NPs) (5.2 and 58.1 nm, respectively) was higher compared to free CUR in PC-3 cells. Unlike free CUR, the NPs also induced apoptosis, with superior effects from the HPMC-CUR-NPs. The HPMC-CUR-NPs had a greater antiproliferative effect and higher cytotoxicity via higher internalization, retention, and apoptosis compared to free CUR in C4-2, DU-145, PC-3, and LNCaP cells (IC_50_: 11.5 ± 4.2–37.4 ± 3.2 µM vs. 7.8 ± 1.78–30.1 ± 1.9 µM). Clearly, the HPMC-CUR-NPs could be potentially useful in prostate cancer treatment. 

In a later study, PLGA-CUR-NPs prepared via nanoprecipitation caused large and extensive vacuoles in C4-2 and DU-145 cells, but fewer and smaller vacuoles in PC-3 cells, which is possibly indicative of lesser NP uptake. Conversely, this was not observed in cells treated with free CUR. The NPs also inhibited cell growth better in vitro (especially at 4 and 6 μM) and reduced tumor volume in mice with C4-2 xenograft tumors better than the free drug without causing systemic toxicity. Additionally, the conjugation of prostate-specific membrane antigen (PSMA) monoclonal antibody to the NPs resulted in improved NP uptake and targeted CUR delivery to PSMA-expressing cells [102].

Rao and colleagues [103] have also prepared thermally-responsive CUR-NPs (EE, 54.3–73.9%) using Pluronic F127 and chitosan via an emulsification–interfacial crosslinking–solvent evaporation–dialysis method. NP size was reduced as temperature was increased from 22 °C (~300 nm) to 37 °C (~22 nm). Mild hyperthermia (43 °C for 1–1.5 h) increased NP uptake, retention, and delivery to the nuclei of PC-3 cells. The IC_50_ of NPs plus hyperthermia was >7-fold lower than that of NPs only, suggesting that mild hyperthermia combined with the CUR-NPs may increase PC-3 cell destruction. However, no in vivo studies were conducted to support these results. 

CUR-loaded, pH-sensitive, redox NPs (PR-CUR-NPs) (35 nm, 82% EE) have been formulated using poly(ethylene glycol)-b-poly [4-(2,2,6,6-tetramethylpiperidine-1-oxyl)aminomethylstyrene]) by the dialysis method [104]. The PR-CUR-NPs improved CUR solubility, suppressed its oxidative degradation, and were significantly more toxic to PC-3 cells compared to free CUR or empty NPs. The PR-CUR-NPs (10 mg/kg, intravenously) also significantly reduced tumor volume in tumor-bearing nude mice compared to free CUR by suppressing oxidative stress.

Polyvinyl alcohol (PVA)-CUR-NPs (46–67.3 nm, −34.6 ± 1.4 to −37 ± 4.3 mV, 71.6–90.3% EE) have been fabricated using the flash nanocomplexation technique via hydrogen bonding interactions [105] without an organic solvent. The cellular uptake and cytotoxicity of the NPs in PC-3 cells was negatively correlated to drug load. Interestingly, free CUR underwent a higher cellular uptake and was more cytotoxic than the NPs. Formulation optimization could possibly improve these findings.

### 4.5. Lipid NPs

In a recent study by Tanaudommongkon and colleagues [106], Miglyol 812 and d-alpha-tocopheryl PEG succinate 1000 (MT) were used to formulate CUR-NPs (138.7 ± 5.4 nm, −24.4 mV, 96.3 ± 6.0% EE) by the nanoemulsion method. The NPs were cytotoxic to docetaxel (DTX)-resistant castration-resistant prostate cancer (CRPC) cells. Compared to free CUR, the CUR-MT-NPs were 5-fold and about 2-fold more cytotoxic to PC-3 and DU145 cells, respectively. The CUR-MT-NPs were also equally cytotoxic to sensitive and resistant cells, similar to the observation for free CUR against PC-3 cells. The CUR-MT-NPs completely overcame the resistance to DTX in both PC-3 and DU145 cells.

## 5. Lung Cancer

### 5.1. Liposomes

Rahman and colleagues [107] fabricated liposomes (420 nm, EE > 66%) containing βCD-CUR complexes with better CUR water solubility than free CUR. The complexes and NPs were prepared by the methanol reflux and thin-film hydration methods, respectively. The median effective dose (EC_50_) values for the CUR-loaded liposomes, free CUR, βCD-CUR, and βCD-C-loaded liposomes were 0.90, 1.5, 2.4, and 2.9 µM, respectively, on A549 cells (*p* < 0.05), clearly showing that increasing the aqueous solubility of CUR may not necessarily correlate with improved cytotoxicity.

### 5.2. Lipid NPs

Wang and colleagues [108] used the sol–gel method to fabricate CUR-loaded solid lipid nanoparticles (SLNs) (20–80 nm, −11.6 mV, 75% EE) using stearic acid, lecithin, and polyoxyethylene (50) stearate. The IC_50_ of the CUR-SLNs (4 μM) against A549 cells was 20-fold lower than that of free CUR. The NPs (200 mg/kg daily, 5 days/week, 19 days) had no effect on body weight, but significantly reduced tumor volume by 65.3% in nude female mice xenografted with A549 cells, compared to 19.5% by free CUR. Additionally, the SLNs significantly increased CUR bioavailability (26.4-fold) in female BALB/c mice and mostly accumulated in the lung and tumor tissues after intraperitoneal administration [108]. 

CUR-loaded cationic lipid NPs (CUR-CLNs) (194.9 ± 7.4 nm, −28.15 ± 2.25 mV, ~98% EE) formulated by the emulsification evaporation-low temperature solidification method also showed better oral pharmacokinetic characteristics (higher bioavailability, higher plasma concentration, and lower clearance) compared to free CUR in rats. Following intravenous administration, the relative bioavailability of the CUR-CLNs to free CUR was 439.76%. The CUR-CLNs also had a better anticancer effect in vitro (Lewis lung cancer, LLC cells; IC_50_ 20.25 μM vs. 39.70 μM) and in vivo (LLC-bearing C57BL/6J mice; tumor growth inhibition rate, ~66 vs. ~39%). The higher anticancer efficacy of the CUR-CLNs was attributed to the increased uptake and higher accumulation in the cells [109].

### 5.3. Gold NPs

Hoshikawa and colleagues [110] have developed PEGylated gold NPs with photothermal effects for CUR delivery. The NPs (<10 mV) were conjugated to CD (α-, β-, and γ-CDs) for CUR encapsulation, with β-CD producing the highest CUR EE%. The average size of the NPs, regardless of the CD used, was 25–35 nm (gold nanocore, ~5 nm). The CUR-CD-Au-NPs were significantly cytotoxic to A549 cells; however, their effect was similar to that of free CUR.

### 5.4. Polymeric NPs

Yin and colleagues [111] have fabricated CUR-NPs using three amphiphilic methoxy PEG (mPEG)–polycaprolactone (PCL) block copolymers via the nanoprecipitation method, with the mPEG10k–PCL30k giving the highest drug loading efficiency and the most sustained drug release profile. The particle size, zeta potential (ZP), and encapsulation efficiency ranges were 102.3 + 11.3 − 140.3 + 14.2 nm, −4.7 + 0.4 to −7.8 + 1.4 mV, and 75.2 + 6.3 − 83.1 + 5.8%, respectively. Reduction of A549 cell viability after 24–72 h of treatment was better with the NPs compared to free CUR, but similar at doses >80 µM. CUR uptake by the cells was also increased by the NPs [111]. 

CUR-coordinated ROS-responsive NPs (163.8 nm, −0.31 mV, 65% EE) have been fabricated using a biocompatible 4-(hydroxymethyl) phenylboronic acid-modified PEG-grafted poly(acrylic acid) polymer (PPH). The NPs improved CUR stability and were potent against A549 cancer cell proliferation in vitro. ROS inhibition with *N*-acetylcysteine resulted in the suppression of the cytotoxic effect of the NPs, which validates the selectivity of PPH-NPs for high-ROS cancer cells. Importantly, the findings showed that CUR release from the NPs was enhanced in the presence of ROS [112].

CUR-containing chitosan NPs (170–200 nm) have been prepared by ionic gelation and evaluated against human non-small cell lung carcinoma (H1299) cells [113]. Toxicity, bioavailability, and chemopreventive efficacy were evaluated in Swiss albino mice after the mice were administered the NPs or free CUR one week before treatment with benzo[a]pyrene (B[a]P), and then on alternative days for up to 4 months. CUR retention in the lungs of the mice was higher for the NPs than for free CUR. One fourth of the NP dose was also more potent in inhibiting B[a]P-induced lung carcinogenesis than free CUR was. Additionally, the CUR-loaded NPs were more effective in reducing nodule size, showing that the NPs can improve the chemopreventive efficacy of CUR against lung cancer.

## 6. Colorectal Cancer (CRC)

### 6.1. Liposomes

Pandelidou and colleagues [114] formulated and evaluated CUR-loaded liposomes (108.0 ± 8.9 nm, 85% EE) for anticancer effect against CRC cells. The liposomes were taken up by HCT116 cells to a greater extent than free CUR, and were subsequently more cytotoxic to HCT116, HCT15, and DLD-1 cells compared to free CUR (IC_50_: < 6 µM vs. 4.5–47.3 µM). The liposomes improved CUR activity against the cells, showing a higher potency against the HCT116 and HCT15 cells. 

In another study, Chen and colleagues [115] formulated CaCO_3_-encapsulated liposomes containing CUR (LCC) (155.3 ± 3.8 nm, −14.2 ± 0.3 mV, 77.67 ± 1.82% EE) with pH-sensitive properties for targeted CUR release by W/O emulsion-mediated film dispersion. The LCC formulation was more cytotoxic to HCT116 cells compared to free CUR or CUR-only liposomes. Similarly, LCC caused a reduction in tumor volume in C57BL/6 mice with colon cancer better than CUR, attributable to enhanced CUR accumulation in the tumors. 

In the aforementioned study by Rahman and colleagues [107], CUR-containing complexes and liposomes were also tested on SW-620 cancer cells, where the EC_50_ values for CUR-loaded liposomes, free CUR, CUR-loaded complexes, and βCD-C-loaded liposomes were 0.96, 1.9, 2.95, and 3.25 µM, respectively. This finding was similar to the trend observed for the lung cancer cells, with the βCD-C complex preparations having the lowest antiproliferative effects, possibly due to the higher aqueous solubility of CUR provided by the complex.

### 6.2. Micelles

Gou and colleagues [116] prepared CUR-containing monomethoxy PEG-PCL micelles (27.3 ± 1.3 nm, 99.16 ± 1.02% EE) by the single-step nanoprecipitation method. Although the micelles were less cytotoxic to C-26 cells than free CUR (IC_50_: 3.95 µg/mL vs. 5.78 µg/mL), the inhibition of angiogenesis was better in an alginate-encapsulated tumor cell assay. Importantly, the micelles (25 mg/kg CUR, intravenously) inhibited colon tumor growth in mice more than the free drug, showing their potential use in CRC management. 

Another type of CUR-loaded micelles prepared with stearic acid-g-chitosan oligosaccharide (CSO-SA) (114.7 ± 16.9 nm, 18.5 ± 0.4 mV, 29.9 ± 2.9% EE) was found to protect CUR from biotransformation and hydrolysis, thereby improving CUR stability. The CUR-CSO-SA micelles showed higher in vitro uptake and 6-fold higher cytotoxicity in CRC cells than free CUR. After 14 days of treatment, the CUR-CSO-SA micelles reduced tumor size and CD44^+^/CD24^+^ cell subpopulation both in vitro and in nude mice [117].

Raveendran and colleagues [118] have used solvent dialysis to prepare a CUR-containing micelles using Pluronic/PCL amphiphilic block copolymer, with PCL, which is hydrophobic, forming the core of the micelles to improve CUR loading. The micelles (195.7 ± 7.3 nm, 17.6 ± 0.4 mV, 72.08 ± 4.29% EE) improved CUR uptake and cytotoxicity in Caco-2 cells.

In a later study, CUR-loaded micelles (27.6 ± 0.7 nm, 0.11 ± 0.34 mV, 96.08 ± 3.23% EE) were also fabricated using monomethyl PEG-PCL and trimethylene carbonate (TMC) via a single-step solid dispersion method. TMC stabilized the micelles by inhibiting PCL crystallization. The micelles increased CUR uptake and cytotoxicity in CT26 cells, and were more effective in suppressing tumor growth in female BALB/c mice (50 mg/kg CUR, intravenously) with fewer toxic effects [119].

Chang and colleagues [120] have also evaluated CUR micelles (34–80 nm, 58–63% EE) prepared using poly(PEG methyl ether methacrylate)-block-poly(styrene) block copolymer in WiDr human colon carcinoma cells. Interestingly, larger micelles were more rapidly endocytosed and exocytosed than the smaller ones were. However, CUR-loaded micelles were better internalized by the cells than unloaded micelles. Additionally, after 72 h of exposure, smaller CUR-loaded micelles remarkably reduced cell proliferation compared to free CUR [120].

### 6.3. Nanogel (NG)

One benefit of NGs is that they confer structural stability to drug delivery systems and protect encapsulated drugs from degradation. Madhusudana Rao and colleagues [121] have used gelatin and acrylamidoglycolic acid to fabricate CUR-loaded pH-sensitive NGs (100 nm, 42–48% EE) by a simple emulsion polymerization technique. The stabilization of NG networks was achieved with glutaraldehyde. The in vitro CUR release was higher at pH 7.4 than at 1.2. Compared to free CUR, the CUR-NGs showed better anticancer activity against HCT116 cells after 48 h of treatment.

Seok and colleagues [122] have also fabricated a hemocompatible NG using hyaluronic acid (HA) cross-linked zein for CUR delivery. The CUR-NG (200–250 nm, 20–40 mV, 94.15% EE) were more cytotoxic to CT26 cancer cells than normal NIH3T3 cells (IC_50_: 37 µg/mL vs. 94 µg/mL) via a higher apoptosis rate. The CUR-NG (3 mg/kg, intravenously) also showed a higher anticancer effect in BALB/c nude mice compared to free CUR [122].

Recently, Borah and colleagues [123] formulated CUR-loaded amylopectin-albumin core–shell NG functionalized with folic acid (FA) (90 nm, −24 mV, 100% EE) that induced early-stage apoptosis of human HT29 cells, whereas free CUR did not. The NG also increased CUR uptake and retention (by 60%) in FA receptor-positive HT29 cells. Additionally, it showed potential for oral delivery as it was resistant to degradation in simulated gastric and intestinal fluids. 

### 6.4. CD-Based NPs

Ndong Ntoutoume and colleagues [101] have formulated CUR-loaded CD/cellulose nanocrystal complexes by ionic interaction (206.8 nm, −29.6 ± 2.7 mV) that improved CUR uptake by HT29 cells and exhibited an anticancer effect that was 3–4 times more effective than CUR alone. These NPs also increased the aqueous solubility of CUR. 

In another study, CUR-NPs (169–338 nm, 17.1 mV, 53.0% EE) fabricated using chitosan, HA, and sulfobutyl-ether-β-CD by ionic gelation also significantly inhibited HT29 cell proliferation. The NPs were better internalized by the cancer cells compared to normal I407 cells. The aqueous solubility of CUR was also improved (3.72 μg/mL to 70 μg/mL) after encapsulation into NPs [124].

### 6.5. Lipid NPs

Chirio and colleagues [125] have fabricated CUR-SLNs (<300 nm, 22.13 ± 3.19 mV, 28–81% EE) by coacervation based on fatty acid precipitation. The highest EEs were obtained with palmitic acid or PVA 9000. Interestingly, the different SLNs prepared, whether loaded with CUR or not, had similar cytotoxic effects against HCT116 cells, which is attributable to low CUR EE and reduced CUR solubility in NPs with higher EEs. 

### 6.6. Gold NPs

Positively charged CUR-Au-NPs (160 ± 20 nm, 18 ± 3 mV) with pH-, radiofrequency- and thermo-responsive properties have been formulated by Sanoj Rejinold and colleagues [126]. The NPs remained in tumors in mice bearing CT26 xenografts for up to 2 weeks. Additionally, the circulation time of CUR in the blood was much longer (up to a week) for the NPs compared to free CUR. Importantly, CUR accumulation was higher in tumors than in other organs, showing the potential of the NPs in targeted treatment of colon cancer.

Alibolandi and colleagues [127] have also formulated CUR-loaded hybrid dendrimer Au-NPs (<10 nm). PEG-Au-poly(amidoamine) (PAMAM) NPs were prepared first and then loaded with CUR. Mucin-1 conjugated aptamer (Apt) increased the uptake and cytotoxicity of the NPs in HT29 and C26 cells. The in vivo antitumor effects (survival rate and tumor growth inhibition) of the NPs (2 mg/kg CUR, intravenously, BALB/c mice) were better than those of free CUR, and were further improved with the Apt-conjugated NPs. 

### 6.7. Polymeric NPs

Chuah and colleagues [128] have formulated CUR-loaded chitosan NPs (340 ± 4.5 nm, 43.7 ± 0.4 mV, 77.44 ± 0.2% EE) to enhance colonic CUR delivery through mucoadhesion. The CUR-NPs had better mucoadhesion properties than the empty NPs. Uptake of the CUR-NPs by HT29 cells was also higher than that of free CUR. Additionally, HT29 cell viability was better reduced after 72 h of treatment and when CUR was loaded into the NPs. 

CUR-NPs have been fabricated with PLGA, soybean lecithin, and DSPE-PEG_2000_-COOH via nanoprecipitation, and functionalized with a ribonucleic acid Apt against epithelial cell adhesion molecule, which is overexpressed on colorectal adenocarcinoma cells, for targeted CUR delivery. The Apt-CUR-NPs (90 ± 1.9 nm, −36.3 ± 4.2 mV, 89.98 ± 3.8% EE) showed 64-fold higher binding and/or internalization by HT29 cells than CUR-NPs functionalized with negative control Apt. The Apt-CUR-NPs also had a higher antiproliferative activity against HT29 colon cells than free CUR and a significantly higher bioavailability and 6-fold longer half-life in male Sprague Dawley rats than the free drug, showing their potential benefit in CRC treatment [129]. 

CUR-loaded polymeric NPs (136 nm, 48 mV, 95% EE) have also been fabricated using chitosan and gum arabic by Udompornmongkol and Chiang [130] via emulsification-solvent diffusion. The NPs were stable in simulated gastrointestinal fluids. Their uptake by HCT116 and HT29 cells, as well as their antiproliferative effect against the cells, were better than those of free CUR, showing the anti-CRC potential of the NPs.

In another study, CUR-NPs prepared by emulsification-diffusion-evaporation using Eudragit E 100 (248.40 ± 3.89 nm, 65.77 ± 3.17% EE) had a 19-fold higher inhibition rate on the growth of Colon-26 cells, had a better oral bioavailability in Wistar rats, and a reduced tumor volume in Colon-26 tumor-bearing BALB/c mice (50 mg/kg CUR, orally, daily for 30 days) as compared to free CUR, showing the potential of the CUR-NPs in CRC management [131].

Silk fibroin (SF) has been used to fabricate CUR-NPs (<100 nm) via solution-enhanced dispersion by the supercritical CO_2_ technique. The IC_50_s of free CUR, CUR-SF-NPs, and fluorouracil against HCT116 cells were 5.339, 4.383, and 0.432 μg/mL, respectively; however, the CUR-SF-NPs had the most superior anticancer effect at CUR concentrations >10 μg/mL [132]. 

Polymeric self-emulsifying NPs (100–180 nm, 64.85 ± 0.12% EE) were formulated by Wadhwa and colleagues [133] via quasi-emulsion solvent diffusion using HPMC acetate succinate. The in vitro release studies showed that up to a 5 h lag time preceded CUR release from the NPs, which approximates the time for cecal arrival following oral intake. Furthermore, the optimized NPs were more cytotoxic to HT29 cells than free CUR (IC_50_: 20.32 µM vs. 28.56 µM), and CUR was successfully delivered to the colon in guinea pigs by the NPs, even though they were not well absorbed in this region.

Alkhader and colleagues [134] have prepared CUR-containing chitosan-pectinate NPs (CUR-CS-PEC-NPs) (206.0 ± 6.6 nm, 32.8 ± 0.5 mV, 64% EE) that are more mucoadhesive at an alkaline pH than at an acidic pH. A very high CUR release (>80%) was achieved in a pectinase-enriched medium (pH 6.4), indicating that the NPs are suited for colon-targeted CUR delivery. In a further study, Alkhader and colleagues [135] found that free CUR and CUR-CS-PEC-NPs inhibited HT29 cell proliferation in a dose- and time-dependent manner. Uptake of free CUR and CUR-CS-PEC-NPs by HT29 cells was comparable; however, the oral bioavailability of CUR in Sprague Dawley rats was significantly higher for the NPs. 

Sabra and colleagues [136] have also formulated CUR-loaded modified citrus pectin-chitosan NPs (MCPC-CUR-NPs; 178 ± 0.896 nm, 35.7 ± 1.41 mV, 69.43% EE) that exhibit mucoadhesive properties using a one-step ionic gelation technique. Similar to the findings of Alkhader and colleagues [134], the MCPC-CUR-NPs exhibited a better mucoadhesive property at pHs of 7.0, 5.5, and 6.25 than at 1.2. Additionally, CUR release over 24 h from the NPs was higher in a 33% (*w*/*v*) cecal medium than in an acidic medium (pH 1.2) (68% versus 18%). In a further study, the MCPC-CUR-NPs showed more toxicity to HT29 and HCT-116 cells compared to unmodified NPs or free CUR, particularly after ≥48 h of treatment. Additionally, pectin modification significantly increased the cellular uptake of the NPs, especially at low CUR concentrations [137]. These results show that MCPC-CUR-NPs may be beneficial in the management of CRC.

## 7. Breast Cancer

### 7.1. Lipid NPs

CUR-loaded transferrin-mediated SLNs (Tf-CUR-SLNs; 206 ± 3.2 nm, 8.21 ± 0.89 mV, 77.27 ± 2.34% EE) prepared by homogenization have been shown to increase CUR photostability and inhibit MCF-7 cell proliferation. Cellular uptake of the Tf-CUR-SLNs was higher than that for free CUR or CUR-SLNs, possibly due to the targeting effect by Tf-CUR-SLNs on MCF-7 cells [138].

Sun and colleagues [139] fabricated CUR-loaded SLNs (152.8 ± 4.7 nm, 90% EE) by high-pressure homogenization, where cell viability was inhibited for a prolonged period, whereas cells treated with free CUR recovered viability after 72 h, confirming the superiority of the NPs. The cellular uptake of the NPs increased from 10 min to 3 h of incubation, then decreased after 3–6 h. On the other hand, uptake of free CUR was rapid in the first 10 min, but decreased sharply afterwards. Additionally, the bioavailability of CUR in Sprague Dawley rats after intravenous administration was 1.25-fold higher from the NPs compared to free CUR. 

Wang and colleagues [140] have also prepared CUR-SLNs by low-temperature solid emulsification using stearic acid and lecithin as lipids. The CUR-SLNs (40 nm, −25.3 ± 1.3 mV, 72.47% EE) induced a higher level of cytotoxicity and apoptosis (IC_50_: 28.42 μM vs. 18.78 μM) and were better taken up by SK-BR-3 breast cancer cells than free CUR. Similar to several of the reported studies on CUR-loaded NPs, no in vivo anticancer evaluations were performed. 

Minafra and colleagues [141] produced CUR-SLNs (302.5 nm, 41.4 ± 4.6 mV, 68.62% EE) with radiosensitizing ability by ethanolic precipitation followed by homogenization. Free CUR and the CUR-SLNs had a comparable cytotoxic effect on MDA-MB-231 breast cancer cells. Irradiation of cancer cells that had accumulated by CUR-SLNs with 2 Gy of photon beam resulted in the protection against radiation-induced oxidative stress and an antitumor effect through the activation of autophagy simultaneously. These findings necessitate further studies on the concomitant use of CUR-SLNs and radiotherapy for treating breast cancer.

### 7.2. NG

Wei and colleagues [142] have prepared a NG loaded with CUR conjugated to cholesteryl-HA (CHA) for targeted CUR delivery to CD44-expressing drug-resistant cancer cells. The NG particles (20 nm) were stable in simulated gastrointestinal fluids and had a 400-fold higher aqueous solubility than pure CUR. The NG was also more cytotoxic to 4T1 cells (IC_50_: 2 μg/mL vs. 5 μg/mL) and inhibited tumor growth in BALB/c mice better than free CUR did. 

### 7.3. Silica NPs

Li and colleagues [143] found that CUR-loaded mesoporous silica NPs (MSNs) modified with HA or polyethyleneimine (PEI)-FA were more cytotoxic to MDA-MB-231 cells at 20–60 µg/mL than free CUR, CUR-MSNs, or CUR-PEI-MSNs were. This was attributed to the affinity of the CUR-PEI-FA-MSNs for FA and CD44 receptors, which are typically overexpressed on MDA-MB-231 cells. Cellular uptake was in the order: CUR-PEI-FA-MSNs > CUR-HA-MSNs > CUR-PEI-MSNs > CUR-MSNs > free CUR. The CUR-PEI-FA-MSNs (8 mg/kg CUR, every 3 days via the tail vein) showed the best antitumor effect in female BALB/c nude, pointing to the importance of suitably surface-modified NPs in cancer management.

### 7.4. ZnO NPs

CUR-loaded phenyl boronic acid (PBA)-conjugated and pH-responsive ZnO NPs (CUR-ZnO-PBA-NPs) (413.63 ± 9.5 nm, −16.4 ± 0.30 mV, 27% EE) have been fabricated by Kundu and colleagues [144]. The uptake of ZnO-PBA-NPs was higher than ZnO-NPs by MCF-7 cells. Furthermore, CUR-ZnO-PBA-NPs were more cytotoxic to MCF-7 cells, accumulated more in tumors, and reduced tumor volume better in Swiss albino mice (10 mg/kg CUR, intravenously on alternate days for 14 days) compared to free CUR. Intriguingly, the CUR-ZnO-PBA-NPs alleviated tumor-induced splenomegaly, which could be valuable in breast cancer patients. 

### 7.5. Hybrid Magnetic-Polymeric NPs

CUR-loaded magnetic alginate/chitosan NPs (CUR-MAC-NPs) (172–199 nm, −28.7 to −34.2 mV, 67.5% EE) prepared via co-precipitation were internalized by MDA-MB-231 3–6 fold higher than free CUR. The CUR-MAC-NPs were also significantly less toxic to normal human dermal fibroblasts. However, in vivo studies are needed to clarify the potential of the CUR-MAC-NPs using an external magnetic field [145].

### 7.6. Human Serum Albumin (HSA) NPs

Saleh and colleagues [146] have formulated CUR-containing HSA-NPs decorated with human epidermal growth factor receptor 2 (HER2) Apt (Apt-CUR-HSA-NPs; 281.1 ± 11.1 nm, −33.3 ± 2.5 mV, 71.3% EE%) through desolvation. The NPs increased the aqueous solubility of CUR 400-fold. Additionally, the presence of Apt increased the cytoplasmic uptake and cytotoxicity of the NPs in HER2-overexpressing SK-BR-3 cells, indicating that the Apt-CUR-HSA-NPs may be a promising treatment for HER2-positive breast cancer. Recently, Hasanpoor and colleagues (2020) similarly fabricated CUR-HSA-NPs and functionalized the particles with programmed death ligand 1 (PDL1) binding peptide, with the peptide-CUR-HSA-NPs (246.5 nm, −24.5 ± 1.5 mV, 77.8% EE) showing a similar trend of uptake and cytotoxicity in high PDL1-expressing MDA-MB-231 cells. Matloubi and Hassan [147] have likewise fabricated CUR-HSA-NPs (220 nm, −7 mV, 70% EE), but without surface functionalization. The cytotoxicity of the NPs was lesser on peripheral blood mononuclear cells, but higher on MCF7 and SK-BR3 cells from 48 h of treatment compared to free CUR. These findings are promising, with regard to improved anticancer effects due to NP surface functionalization. However, further studies in animal models are needed to show their potential in breast cancer management.

### 7.7. Magnetic NPs

Ashkbar and colleagues [148] found that Fe_3_O_4_-SiO_2_-CUR-NPs (20–60 nm, −57.5 mV) combined with photodynamic therapy (PDT) and photothermal therapy (PTT) produced the highest reduction of 4T1 tumor volume in female BALB/c mice compared to the NPs only, free CUR plus PDT, NPs plus PTT, or PDT plus PTT. Importantly, tumor volume was decreased by 58% by the NPs alone compared to the untreated group. These show the benefit of the CUR-NPs and, even more significantly, the potential of the triple therapy in breast cancer management.

### 7.8. Polymeric NPs

Khan and colleagues [149] fabricated CUR-PLGA NPs (265–606 nm, −3.1 to −13.7 mV, >50% EE) by solvent evaporation and tested them on MDA-MB-231 cells, whereby a 10-fold increase in solubility and 3-fold increase in anticancer activity compared to CUR was observed. Sampath and colleagues [150] have investigated the use of tocopherol PEG 1000 succinate (TPGS, as emulsifier) with different capping agents (chitosan, dextran, and PEG) to fabricate CUR-PLGA-NPs by emulsion solvent evaporation (<200 nm, 7.94–40.47 mV, 82–89% EE). All NPs produced reduced MCF-7 cell proliferation better than free CUR did, with the highest cellular uptake observed when TPGS and dextran were used. 

CUR-loaded poly-glycerol-malic acid-dodecanedioic acid NPs (110–218 nm, −17.5 to −18.9 mV, 75–81% EE) fabricated via nanoprecipitation have shown a higher antiproliferative effect against MCF-7 and MDA-MB 231 cells compared to free CUR. Interestingly, the cytotoxicity of free CUR decreased with increasing incubation periods, whereas the reverse was observed for the NPs after 7 days [151].

### 7.9. Phospholipid-Polymeric NPs

CUR-loaded phospholipid NPs conjugated with EGF have been formulated via thin-film hydration by Jung and colleagues [152]. The EGF-CUR-NPs (229.3 ± 6.0 nm, 63.3% EE) exhibited a dose-dependent suppression of MDA-MB-468 TNBC cell survival and were more cytotoxic than free CUR or CUR-only NPs. The EGF-CUR-NPs (10 mg/kg, intraperitoneally, three times weekly, total of 8 injections) also suppressed tumor growth in mice bearing MDA-MB-468 tumors better compared to empty or CUR-only NPs, showing that they may be beneficial for treating TNBCs with an overexpression of EGF receptors.

### 7.10. Polymer-Coated Gold NPs

FA-functionalized CUR-Au-PVP-NPs (358.7 nm, −12.5 mV) prepared by layer-by-layer assembly have been shown to be cytotoxic to MDA-MB-231, MCF-7, and 4T1 cancer cells, but not to normal L929 and MCF-10A cells at CUR concentrations of <100 µg/mL. The FA-CUR-Au-PVP NPs (10 mg/kg, intratumorally, 2 weeks) inhibited tumor growth in BALB/c mice bearing 4T1 tumors more than free CUR [153].

### 7.11. Radiolabeled NPs

Huang and colleagues [154] used membrane dialysis to fabricate self-assembled 99mTc-radiolabeled CUR-NPs with HA-cholesteryl hemisuccinate conjugates and D-a-TPGS. The NPs (144 nm, −21.25 ± 1.66 mV, 84.0 ± 5.0% EE) inhibited 4T1 cell growth by two-fold compared CUR (IC_50_ values: 38 μg/mL vs. 77 μg/mL). Tumor growth in tumor-bearing BALB/c mice was also inhibited by the NPs (50 mg/kg, every 2 days, total of 5 injections) without toxicities to major organs; however, the in vivo effect of free CUR was not determined.

### 7.12. NG

Setayesh and colleagues [155] grafted octadecylamine (ODA) to chondroitin sulfate (CS) to form a CS-ODA conjugate that was used to prepare CUR-loaded NG (311 ± 20.29 nm, −13.25 ± 0.35 mV, 79.56 ± 5.56% EE). Interestingly, the CS-ODA-NG and free CUR were equally cytotoxic to MCF-7 cells after ≥48 h of incubation; however, cellular uptake of the NG was higher.

## 8. Oral, Cervical, Ovarian, and Pancreatic Cancers

Reported studies on the evaluation of CUR nanoformulations for oral, cervical, ovarian, and pancreatic cancers have been summarized in Table 1.

## 9. Synergistic Effect of CUR-NPs with Other Anticancer Drugs 

Combination drug treatment is a popular strategy that can be beneficial in overcoming drug resistance and dose-limiting toxicity, which are typical concerns associated with the management of cancer [179]. Some studies have shown that NPs containing CUR in addition to other drugs can have synergistic anticancer effects. For instance, NPs co-loaded with CUR and doxorubicin have been shown to be effective against lung cancer [180]. In other studies, NPs containing CUR and paclitaxel [181], methotrexate [182], GANT61 [183], or doxorubicin [184] have been shown to be effective against breast cancer. It has also been found that CUR and docetaxel [185] or CUR and cabazitaxel [186] have synergistic effects against prostate cancer. Finally, it has been revealed that CUR and oxaliplatin [187], 5-fluorouracil [188], camptothecin [189,190], chrysin [191], doxorubicin [192,193], or cetuximab [194] may synergistically inhibit the growth of colon cancer cells. From the foregoing, it is possible that the abovementioned cancers may be better managed with NPs loaded with both CUR and the other anticancer drugs. However, clinical trials are required to clarify the effectiveness of these proposed combination therapies.

## 10. Clinical Trials Conducted on CUR-NPs

Pharmaceutical formulations that show promise in preclinical studies must be evaluated in clinical trials, because preclinical findings may not be necessarily be reflected in humans. 

There is only a handful of clinical trials (ongoing or completed) covering the therapeutic evaluation of CUR-NPs, with trials related to CUR-NPs for cancer management being even more scarce. A clinical trial has been conducted on a CUR-NP formulation called THERACURMIN, in which six healthy volunteers were orally administered two doses (150 and 210 mg) of the preparation. The trial showed a high CUR bioavailability following administration to human subjects. However, the subjects were not administered free CUR [195]. It was also found from a randomized, placebo-controlled, double-blind phase I dose escalation study performed in healthy participants (n = 50, male and female) that short-term administration of intravenous liposomal CUR (<0.2 µm; dose, 120 mg/m^2^) may be safe but can cause changes in red blood cell morphology [196].

A clinical trial on the use of plant exosomes (50–100 nm) to deliver CUR to colon tumors and normal colon tissue started in January 2011. Subjects are being administered CUR tablets, CUR conjugated with plant exosomes, or no treatment, with the primary outcome measure being CUR concentration in normal and cancerous tissue. The study is ongoing and therefore no outcomes have been published yet (NCT number: NCT01294072, 2011).

CUR-containing nanostructured lipid particles (100 mg p.o. bid daily) have been evaluated in an open-label phase II clinical trial (50 colorectal cancer patients with unresectable metastasis) in addition to standard chemotherapy treatment (Avastin/FOLFIRI [folinic acid, fluorouracil, irinotecan]). The primary end point was to evaluate progression-free survival (time frame, 2 years) (NCT number: NCT02439385, 2015).

Saadipoor and colleagues [197] have conducted a randomized controlled trial to assess the benefit of CUR-loaded nanomicelles (10 nm, ∼100% EE) in radiation-induced proctitis in prostate cancer patients undergoing radiotherapy. Unfortunately, the CUR formulation was not found to be efficacious in the patients.

From the foregoing, it is very evident that more evaluations of CUR nanoformulations in human subjects are required. This is important because several studies have shown promising anticancer benefits of some CUR-NPs in vitro and in animal models.

## 11. Conclusions

CUR has received extensive interest for its diverse health benefits in general and its anticancer effects in particular. It has been proposed as a natural and effective anticancer agent with mild to no side effects. A plethora of studies have shown multiple anticancer mechanisms of CUR and the synergistic effects of combining CUR with other anticancer agents. Since poor bioavailability is the main obstacle in the use of CUR for cancer treatment, research has been devoted towards fabricating advanced drug delivery systems to improve CUR stability and bioavailability. A good number of in vitro, in vivo, and clinical studies have shown that CUR has an anticancer effect, and that NPs improve the oral bioavailability, stability, and targeted delivery of CUR, which could make CUR-NPs useful in cancer management.

## Figures and Tables

**Table 1:** Summary of details from studies on CUR nanoformulations for ovarian, oral, cervical, and pancreatic cancers.

Study Authors (Reference)	NP Type and Details	Cancer Cell Line/Cytotoxicity (IC_50_ of NPs vs. Free CUR)	Cellular Uptake of CUR-NPs vs. Free CUR	Animal Model	Treatment Regimen	*In Vivo* Antitumor Activity	Toxicity
Ovarian cancer							
[156]	Polymeric NPs (PLGA, PVA, and poly-L-lysine) PM: Nanoprecipitation SZ: ~72 nm ZP: N/I EE: N/I	A2780CP (resistant to cisplatin) paired cells CUR-NPs inhibit cell proliferation compared to blank NPs IC_50_: N/I	N/D	N/D	N/D	N/D	N/D
[157]	Nanostructured lipid carriers (Compritol 888 ATO, Captex 355 EP/NF, Miglyol 812) PM: Precipitation SZ: 100–160 nm ZP: N/I EE: 40–100%	A2780S cells (cisplatin-sensitive) NPs: 21.2 ± 3.5 μM Free CUR: 22.2 ± 1.8 μM A2780CP cells (cisplatin- resistant) NPs: 19.0 ± 1.4 μM Free CUR: 20.2 ± 2.5 μM	Similar	N/D	N/D	N/D	N/D
[158]	Polymeric NPs (PLGA, PVA) PM: Emulsion–diffusion–evaporation Non-lyophilized NPs SZ: 203.6 ± 7.8 nm ZP: −5.24 ± 0.86 mV EE: 80.4 ± 10.6% Lyophilized NPs SZ: 201.8 ± 6.0 nm ZP: −5.43 ± 0.67 mV EE: N/I	SK-OV-3 cells ↑ for NPs (measured as amount of ROS generated)	↑ for NPs (with or without irradiation from LED-based photodynamic therapy)	N/D	N/D	N/D	N/D
Oral cancer							
[159]	Polymeric NPs (PLGA and PVA) PM: Single emulsion solvent evaporation SZ: 180 nm ZP: N/I EE: N/I	CAL27-cisplatin resistant cells IC_50_: N/I >80 µM for normal human gingival fibroblasts cells oral keratinocyte cells	N/D for free CUR	N/D	N/D	N/D	N/D
[160]	Silica NPs (Vinyltriethoxysilane, 3-aminopropyl-trimethoxysilane) PM: N/I SZ: ~32 nm ZP: −39 ± 1.0 mV EE: N/I	Human squamous cell carcinoma (4451) cells IC_50_: N/I	↑ for NPs	N/D	N/D	N/D	N/D
[161]	Chitosan-coated PCL NPs PM: Nanoprecipitation SZ: 115.3–127.3 nm ZP: 23.5–40.1 mV EE: >99%	SCC-9 cells 24 h NPs: 271.5 ± 1.17 µM Free CUR: 93.40 ± 4.26 µM 48 h NPs: 260.3 ± 8.35 µM Free CUR: 75.21 ± 3.25 µM 72 h NPs: 92.04 ± 1.53 µM Free CUR: 17.46 ± 1.20 µM (*p* < 0.05 in each instance) Free CUR was more cytotoxic than the NPs	N/D	N/D	N/D	N/D	N/D
Cervical cancer							
[27]	Polymeric NPs (PLGA; co-polymerization ratios 50:50 and 75:25, lactic/glycolic) PM: Single emulsion (solvent evaporation) SZ: 100–200 nm ZP: N/I EE: 74.73–90.03%	HeLa cells Comparable cytotoxicity	↑ for NPs	ND	ND	ND	ND
[162]	NG (FA-conjugated cross-linked polymeric NPs) (acrylic acid, PEG diacrylate, FA) PM: Inverse emulsion polymerization SZ: 160–190 nm ZP: N/I EE: 61.2 ± 1.2%	HeLa cells ↑ for NPs IC_50_: N/I	↑ for NPs	ND	ND	ND	ND
[163]	Liposomes (soybean lecithin and cholesterol, Montanov82^®^, and/or DDAB) PM: Thin film hydration method CUR-NPs (with Montanov82^®^) SZ: 161.5 ± 0.8 nm ZP: −1.4 ± 0.8 mV EE: 63.9 ± 3.8% CUR-NPs (with cholesterol) SZ: 161.8 ± 0.4 nm ZP: −0.1 ± 0.1 mV EE: 70.6 ± 0.5% CUR-NPs (with Montanov82^®^ and DDAB) SZ: 252.4 ± 5.3 nm ZP: 28.8 ± 1.0 mV EE: 34.7 ± 0.3% CUR-NPs (with cholesterol and DDAB) SZ: 219.5 ± 9.3 nm ZP: 27.7 ± 0.9 mV EE: 68.9 ± 0.6%	HeLa cells ↑ for NPs than free CUR NPs: N/I Free CUR: 21 µM SiHa cells ↑ for NPs than free CUR NPs: N/I Free CUR: 16 µM For both cells, cytotoxicity ↑ for NPs containing DDAB	N/I	ND	ND	ND	ND
[164]	Micelles (*N*-benzyl-*N,O*-succinyl chitosan synthesized from chitosan by successive reductive *N*-benzylation, and *N,O*-succinylation) PM: Dialysis method SZ: 80 ± 4.0–97 ± 5.0 nm ZP: −27.1 ± 1.4 to −29.2 ± 1.4 mV EE: 7.57 ± 0.01–38.30 ± 5.70%	HeLa cells NPs: 4.34 ± 0.12 µM (4.7-fold ↓) Free CUR: 21.17 ± 1.80 µM SiHa cells NPs: 4.34 ± 0.12 µM (3.6-fold ↓) Free CUR: 16.28 ± 1.34 µM C33A cells NPs: 4.34 ± 0.12 µM (12.2-fold ↓) Free CUR: 54.29 ± 3.62 µM	Significantly ↑ for all NPs	-	-	-	-
[165]	Polymeric NPs (chitosan, sodium tripolyphosphate) PM: Ionic gelation SZ: 197 ± 16.8 nm ZP: 71 ± 6.4 mV EE: ~85%	SiHa cells NPs: 97.27 µg/mL HeLa cells NPs: 88.41 µg/mL CasKi cells NPs: 81.48 µg/mL C33A cells NPs: 95.46 µg/mL IC_50_: Values at 72 h Value N/I for free CUR	N/I Among cells, NP uptake over 25 h was CasKi > C33A > HeLa > SiHa	-	-	-	-
[166]	Polymeric NPs (PLGA, PVA, poly(l-lysine)) PM, SZ, ZP, EE: N/I	Caski cells and SiHa cells IC_50_: N/I ↑ cytotoxicity for NPs	↑ for NPs	Female NOD scid gamma mice Caski cells (4 × 10^6^ cells, injection into cervix) TV: ~200 mm^3^ (maximum tumor burden allowed post treatment: 1100 mm^3^)	100 μg intra-tumoral injection	Changes in TV NPs: 637 ± 68 mm^3^ Free CUR: 816 ± 94 mm^3^	N/I
[167]	Polymeric NPs (Chitosan, alginate, sodium tripolyphosphate) PM: Ultrasonic-assisted method SZ: ~50 nm ZP: N/I EE: 70%	HeLa cells IC_50_: N/I ↑ cytotoxicity for NPs	N/I	ND	ND	ND	ND
[168]	Micelles (Pectin) PM: Self-assembly method SZ: 70–190 nm ZP: N/I EE: N/I	HeLa cells NPs: 14.1 ± 3.0 µM Free CUR: 40.9 ± 2.6	N/I	ND	ND	ND	ND
[169]	Silica/titania mesoporous NPs (coated with polyethylenimine-FA) PM: Hydrolysis, condensation reactions, and surface functionalization. Drug loading (solvent deposition) SZ: 173 ± 15 nm ZP: N/I EE: 43.36 ± 0.32%	HeLa cells ↑ cytotoxicity for NPs (synergetic chemo-sonodynamic therapy observed)	N/I	ND	ND	ND	ND
[170]	Liposomes (DSPE, PEG_2000_, FA, SPC, cholesterol) PM: Thin-film hydration SZ: 112.3 ± 4.6 nm ZP: −15.3 ± 1.4 mV EE: 87.6%	HeLa cells NPs: 0.82 µg/mL Free CUR: 1.47 µg/mL	↑ NPs-66.4 ± 6.2% Free CUR-5.7 ± 1.6%	Female BALB/c mice (~5 × 10^6^ cells in 100 μL PBS, lower right flank) TV: 100–150 mm^3^	25 mg/kg CUR, on alternate days for three weeks	Final TV NPs: 77.3 ± 56.5 mm^3^ Free CUR: 634.3 ± 67.4 mm^3^	No obvious acute toxicity
[171]	Nano-niosomes (Fe_3_O_4_, PLGA, PEG, FA) PM: Double emulsion method (W/O/W) and vacuum drying SZ: 190.4 ± 5.3 nm ZP: N/I EE: 86.46%	HeLa229 cells IC_50_: N/I	↑	-	-	-	-
[172]	Micelles PM: Co-assembly of CUR and cystine/lysine-bridged peptide (CBP/LBP) SZ: ~250 nm ZP: N/I EE: 63.44%	HeLa cells ↑ cytotoxicity for NPs (but higher with the CBP) IC_50_: N/I	↑ for NPs (but higher with the CBP)	Female BALB/c nude mice bearing HeLa cells Treatment was started on the 10th day when the tumor volume reached 100 mm3	2.5 mg/kg intravenous injection into tail, every three days for 14 days	TGI NPs (CBP): 69.12% NPs (LBP): 10.66% Free CUR: 36.14%	NPs: Minimal effects on healthy tissues Free CUR: Apoptosis in liver, kidney, spleen All formulations: No significant Effect on body weight
Pancreatic cancer							
[173]	Polymeric micelles (methoxy(polyethylene glycol), PCL) PM: Modified dialysis SZ: 110 ± 6.4 nm ZP: −16 ± 2.77 mV EE: 57.6 ± 1.23%	PANC-1 cells NPs: 22.8 µM Free CUR: 24.75 µM MiaPaCa-2 cells NPs: 13.85 µM Free CUR: 14.96 µM	Test performed on PANC-1 cells only At 10 µM NP uptake 2.95-fold ↑ At 30 µM NP uptake 1.88-fold ↑	-	-	-	-
[174]	Magnetic NPs (Fe(III) chloride hexahydrate (99%), Fe(II) chloride tetrahydrate, CD, Pluronic F-127) PM: N/I SZ: 109 nm ZP: −0.99 mV EE: N/I	HPAF-II and PANC-1 human pancreatic cancer cell lines IC_50_: N/I Similar cytotoxicity between NPs and free CUR to both cell lines	↑ Similar uptake by both cell lines (54.06% vs. 53.86%)	Male athymic nude (nu/nu) mice Inoculated subcutaneously, left flank (5 × 10^6^ HPAF-II cells)	13th day after inoculation Intratumoral administration, 20 μg CUR in 100 μL vehicle Animals sacrificed at the end of treatment or when TV = 1000 mm^3^	TV ↓ by NPs more than by free CUR	N/D
[175]	Self-assembled casein (sodium caseinate) NPs PM: Self-assembly SZ: 104–213 nm ZP: −37.63 mV to −39.07 mV EE: 70% to ∼100%	BxPC3 cells NPs: 25.3 µg/mL Free CUR: 29.4 µg/mL	↑	-	-	-	-
[142]	NG (Cholesteryl-HA) PM: N/I SZ: 29.2 ± 5.4 nm ZP: −38.4 ± 3.9 mV EE: N/I	MiaPaCa-2 cells NPs: 9 μg/mL Free CUR: 18 μg/mL	N/I	Female nu-nu mice (5 × 10^6^ cells, subcutaneous injection, right flank)	10th day after inoculation Intraperitoneal injection 6 mg/kg CUR twice every week	NG ↓ TV 5-fold vs free CUR by day 49	No significant weight loss
[176]	Polymeric NPs (Chitosan, PEG, PLGA) PM: Emulsion solvent evaporation SZ: 264 nm ZP: 19.1 mV EE: 60%	PANC-1 cells NPs: 14.2 ± 4.6 μM Free CUR: 28 ± 4.1 μM MiaPaca-2 cells NPs: 6.1 ± 0.6 μM Free CUR: 20.3 ± 1.1 μM	PANC-1 cells 6.7-fold ↑ for NPs MiaPaca-2 cells 7.5-fold ↑ for NPs	-	-	-	-
[177]	Chitosan-coated lipid NPs (chitosan, stearoyl chloride, cholesterol) PM: Cold dilution of microemulsion SZ: 190.6 ± 1.5 nm ZP: 2.10 ± 0.51 mV EE: 73.4 ± 0.3%	PANC-1 cell lines IC_50_: N/I ↑ cytotoxicity of NPs at 5 and 10 µM CUR concentration	-	-	-	-	-
[178]	SLNs (trilaurin) PM: Cold dilution of microemulsion SZ: ∼200 nm ZP: −10.06 ± 2.66 mV EE: 75 ± 1.0%	CFPAC-1 and PANC-1 cells IC_50_: N/I ↑ cytotoxicity of NPs	-	-	-	-	-

Abbreviations: CD, cyclodextrin; CUR, curcumin; EE, encapsulation efficiency; FA, folic acid; HA, hyaluronic acid; IC_50_: half-maximal inhibitory concentration; N/D, not determined; N/I, not indicated; NG, nanogel; NP, nanoparticle; PCL, polycaprolactone; PEG, polyethylene glycol; PM, preparation method; ROS, reactive oxygen species; SZ, size; TGI, tumor growth inhibition; TV, tumor volume; ZP, zeta potential.
